# Methane Production and Consumption in Loess Soil at Different Slope Position

**DOI:** 10.1100/2012/620270

**Published:** 2012-05-02

**Authors:** Małgorzata Brzezińska, Magdalena Nosalewicz, Marek Pasztelan, Teresa Włodarczyk

**Affiliations:** Institute of Agrophysics, Polish Academy of Sciences, Ulica Doświadczalna 4, 20290 Lublin, Poland

## Abstract

Methane (CH_4_) production and consumption and soil respiration in loess soils collected from summit (Top), back slope (Middle), and slope bottom (Bottom) positions were assessed in laboratory incubations. The CH_4_ production potential was determined under conditions which can occur in the field (relatively short-term flooding periods with initially ambient O_2_ concentrations), and the CH_4_ oxidation potential was estimated in wet soils enriched with CH_4_. None of the soils tested in this study emitted a significant amount of CH_4_. In fact, the Middle and Bottom soils, especially at the depth of 20–40 cm, were a consistent sink of methane. Soils collected at different slope positions significantly differed in their methanogenic, methanotrophic, and respiration activities. In comparison with the Top position (as reference soil), methane production and both CO_2_ production and O_2_ consumption under flooding were significantly stimulated in the soil from the Middle slope position (*P* < 0.001), while they were reduced in the Bottom soil (not significantly, by 6 to 57%). All upper soils (0–20 cm) completely oxidized the added methane (5 kPa) during 9–11 days of incubation. Soils collected from the 20–40 cm at the Middle and Bottom slope positions, however, consumed significantly more CH_4_ than the Top soil (*P* < 0.001).

## 1. Introduction

Methane (CH_4_) is the most abundant hydrocarbon in the atmosphere, and it is an important greenhouse gas, which so far has contributed to an estimated 18–20% [1, 2] of postindustrial global warming. Methane has environmental impacts beyond those of a direct greenhouse gas, through atmospheric chemistry that enhances the abundance of tropospheric ozone (O_3_) and decreases that of hydroxyl radicals (OH) and hence the atmospheric lifetime of many other pollutants [3]. The atmospheric CH_4_ concentration has risen from the background level from 700 to 1782 ppb in 2006, and the growth rate in CH_4_ concentration was changing considerably; the very large and interannual variations in CH_4_ concentration remain unexplained and present an important challenge to the research community [4, 5]. Estimated surface CH_4_ emissions reach 643 Tg year^−1^ [3]. Oxidation of atmospheric methane by well-drained soils accounts for about 10% [6] or 6% [4] of the global methane sink, that is about 30 Tg CH4 per year. Other CH_4_ sinks are the stratosphere (40 Tg year^−1^) and tropospheric OH (445 Tg year^−1^) [4].

 Most methane on Earth is produced by Archaea through methanogenesis, the final step in fermentation of organic matter, which takes place in rice fields, the guts of animals, soils, wetlands, and landfills, as well as in freshwater and marine sediments. As a simple assumption, about 10–20% of reactive organic material buried in soils and sediments is converted to methane [1]. The potential impact of methane on future global warming and an important role of soils in sorption of this gas have led to many terrestrial studies of methods and techniques to quantify CH_4_ flux at the soil-atmosphere interface [7]. Numerous experimental data on emission of greenhouse gases are used in modelling of the local and global gas emissions, while some models were developed to determine abatement strategies to meet restrictions on emission and/or deposition levels at the least cost [8].

Soil saturation with water has dramatic consequences for gas diffusion processes in soil (as gases diffuse 10,000 faster in air than in water). Consequently, one of the main effects of flooding is a lower pool of available O_2_ [9, 10] and a several-fold change in the activity of the oxidoreductases—intracellular enzymes involved in the oxidative metabolism of soil microorganisms [11]. Conventional knowledge states that water-saturated systems like wetlands (swamps, marshes) and paddy soils (rice fields) are net contributors of CH_4_ to the atmosphere, whereas upland soils (with the exception of landfills) are generally sinks for CH_4_ [12]. However, significant methane emission from field soils may also occur after normal precipitation if the soils remain saturated for a long enough period, since water occupation of soil voids may cause oxygen deficiency and development of reducing conditions. Even in unsaturated conditions, there may be anaerobic microsites capable of evolving methane. Little is known, however, about methane emission when usually well-drained soils become flooded for a short period [13]. In fact, soils can act as a source and a sink for CH_4_, depending on their air-water conditions [7, 14].

Soil properties are a product of soil-forming factors including landscape variability, agroecosystem management, and climatic factors. Numerous studies were performed to measure the effect of landscape position and land management on physical, chemical and biological soil properties [15–21]. Soils developed from loess are fertile and show high erodibility [22]. Soil erosion results in heavy differentiation of a soil cover with natural pedons being reduced or overbuilt. Both eroded and colluvial soils differ from uneroded soils not only in morphological features but also in particle-size and pore distributions, organic matter content and plant nutrients, water retention, and bulk density [23]. Loess soils are among the most susceptible to the drop in redox potential under anaerobic conditions, which is followed by a rapid reduction of the oxidized inorganic soil components [24]. In consequence, periodical soil hypoxia changes soil respiration, which plays a fundamental role in the metabolism of the soil biota and promotes development of methanogenic microorganisms.

The objective of this study was to compare the CH_4_ production and CH_4_ consumption in slightly eroded loess soils taken at the summit, back slope, and bottom of a hill. The experiment was performed in laboratory under controlled temperature and air-water conditions. Initially, ambient O_2_ concentrations were present in both flooded and wet soil incubations. Our intention was to determine the soil potential for methane production under conditions which occur in field (relatively short-term flooding periods for methanogenic activity) and for methane oxidation (soil enriched with CH_4_).

## 2. Materials and Methods

### 2.1. Site and Soil Description

A loessial agricultural basin of the Ciemięga River (near Lublin, south-east part of Poland) is a region of the water erosion risk, including sediment transport and nutrient runoff, and is under intensive agricultural use [24, 25]. Soil samples were collected near Baszki village from two depths (0–20 cm and 20–40 cm) and three slope positions: at the summit (Top), back slope (Middle), and slope bottom (Bottom).

The slope is about 15 m high and 60 m long and is covered by natural grass vegetation; it is at the distance of about 150 m from the river. The annual precipitation in this region is 570 mm, and the average annual temperature is +7.5°C [25]. The basic characteristic of the tested brown loess soil (Eutric Cambisol) is shown in Table 1.

### 2.2. Incubation Experiment

For methanogenic activity measurements, 20 g portions of air-dry soils were placed into 60 cm^3^ glass vessels and flooded with 15 cm^3^ of distilled water. All the vessels were tightly closed with rubber stoppers and aluminium caps, and the flooded soils were incubated at 25°C for 28 days. 

For methanotrophic activity measurements, 10 g portions of air-dried soils were placed into 60 cm^3^ glass vessels and 5 cm^3^ of distilled water was added. All the vessels were tightly closed with rubber stoppers and aluminium caps, and wet soils were enriched with 5% (v/v) CH_4_ (5 kPa). The soil samples were incubated at 20°C for 21 days. 

Initially, ambient O_2_ concentrations were present in both incubations (20.5% v/v). Our intention was to determine the potential of soils for methane production under field conditions, with relatively short-term flooding periods, and for methane oxidation after soil enrichment with CH_4_. 

### 2.3. Methods

The concentrations of gases in the headspace were measured with gas chromatographs Shimadzu GC-14B and GC-14A (Japan) equipped with a flame ionization detector (FID) and a thermal conductivity detector (TCD), respectively. Methane was detected by the FID detector at 150°C. The gas components were separated on a column packed with a Porapak Q maintained at 80°C, and the temperature of the injector was 150°C. Carbon dioxide and O_2_ were detected by TCD with the use of two 2 m columns (3.2 diameter), one packed with Porapak Q (for CO_2_) and the other packed with Molecular Sieve 5A (for O_2_) with He as a carrier gas flowing at a rate of 40 cm^3^ min^−1^. The temperatures of the column and detector were 40°C and 60°C, respectively. The detector responses were calibrated using certified gas standards (Air Products) containing 20.9% O_2_ in N_2_ and 4% CH_4_ or 1% CO_2_ in He [26, 27]. 

Soil properties were determined by standard methods. The organic (C_org_) and inorganic (C_inorg_) carbon was determined using a TOC-VCPH analyzer (Shimadzu, Japan). The particle size distribution was measured with the laser diffraction method [28]. All measurements were done in triplicate, and the results were expressed on an oven-dry weight basis (105°C, 24 h). 

### 2.4. Calculations

The concentrations of gases were corrected for solubility in water by using published values of the Bunsen absorption coefficient [29]. The rates of methane production and consumption were calculated by linear regression of increases and decreases, respectively, in CH_4_ concentrations against incubation time, using at least three consecutive measurements with a regression coefficient (*r* ^2^) of >0.9, and expressed in mg CH_4_-C per kg of oven-dry soil per day [30, 31]. The total cumulative CH_4_ production and CH_4_ consumption were determined in each sample by the difference in the headspace CH_4_ concentration at the beginning and end of the assay period [32]. The rates of CH_4_ production and consumption and the total amount of CH_4_ produced and consumed were used as a measure of the methanogenic and methanotrophic activity (potential), respectively. Similarly, the total amounts of CO_2_ produced and O_2_ consumed were calculated to describe the respiration activity (and expressed as mg CO_2_-C kg^−1^ and O_2_% (v/v), resp.). Final amounts of CH_4_, CO_2_, and O_2_ were assessed by Student's “*t*” test to determine the significance of the differences in gas production or consumption between soils. Correlations between total gases produced or consumed over time, and organic carbon in soils collected from different slope positions were tested with regression analysis. 

## 3. Results

### 3.1. Methanogenic Activity of Soils from Different Slope Positions

**Figure 1:**
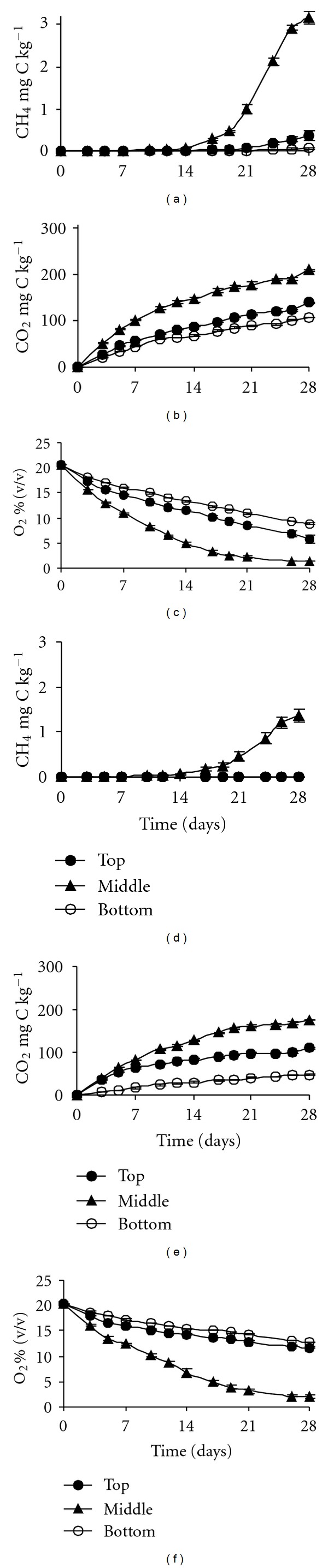
Changes of CH_4_, CO_2_ and O_2_ over time in loess soils collected from three slope positions and incubated under flooding (methanogenic potential). Top: summit, Middle: back slope, Bottom: bottom of the slope. Upper graphs (a–c) upper soil depth of 0–20 cm; lower graphs (d–f) lower soil depth of 20–40 cm. (a) and (d) cumulative CH_4_ production; (b) and (e) cumulative CO_2_ production; (c) and (f) changes in O_2_ in the headspace. Points represent triplicate-means with standard error.

Position of soil in the slope strongly affected the capacity of CH_4_ production. Methane was produced in flooded soils after a 17-day lag (Figure 1). The highest methanogenic activity was observed in the Middle soil. During 28-day incubation, the upper 0–20 cm soil evolved 3.17 mg CH_4_-C kg^−1^ soil at a rate of 0.304 mg CH_4_-C kg^−1^ d^−1^ (Figure 1(a), Table 2). Soil sampled at the Top position produced only 0.359 mg CH_4_-C kg^−1^, while the Bottom soil evolved even less than 0.06 mg CH_4_-C kg^−1^. 

Deeper soil layers (20–40 cm) showed significantly lower methanogenic activity (*P* < 0.001) with the highest production in the soil from the Middle position 1.35 mg CH_4_-C kg^−1^ and much lower in the other soils: less than 0.002 mg CH_4_-C kg^−1^ (Figure 1(d)). 

The tested soils showed relatively large differences in their respiration under flooding. Both CO_2_ evolution and O_2_ consumption were more intensive in the upper rather than deeper soil layers (Figures 1(b) and 1(c)). The Middle soil produced 207.6 mg CO_2_-C kg^−1^, and consumed 19.14% (v/v). At the beginning of CH_4_ evolution in this soil after 17 days of incubation, there was only 3.21% (v/v) O_2_ left in the headspace. At the end of incubation, O_2_ was hardly depleted (1.36% v/v in the headspace). The Top and Bottom soils consumed 14.6 and 11.6% (v/v) O_2_, respectively, which yielded the final O_2_ concentration in the headspace of 5.88 and 8.85% (v/v), respectively. 

The subsurface-flooded soils showed some lower respiration (Figures 1(e) and 1(f)). An exception was the Middle soil, which produced as much as 174.6 mg CO_2_-C kg^−1^, while it consumed 18.5% (v/v) O_2_ (2% v/v O_2_ left in the headspace). The other soils followed the tendency observed in CH_4_ production; thus they evolved less CO_2_ and consumed less O_2_ (<111 mg CO_2_-C kg^−1^ and <8.8% (v/v), resp.). In most cases, the CO_2_ produced and O_2_ consumed in the course of methanogenic incubation in the Middle and Bottom soils significantly differed from those in Top soil (Table 2). 

### 3.2. Methanotrophic Activity of Soils from Different Slope Positions

**Figure 2:**
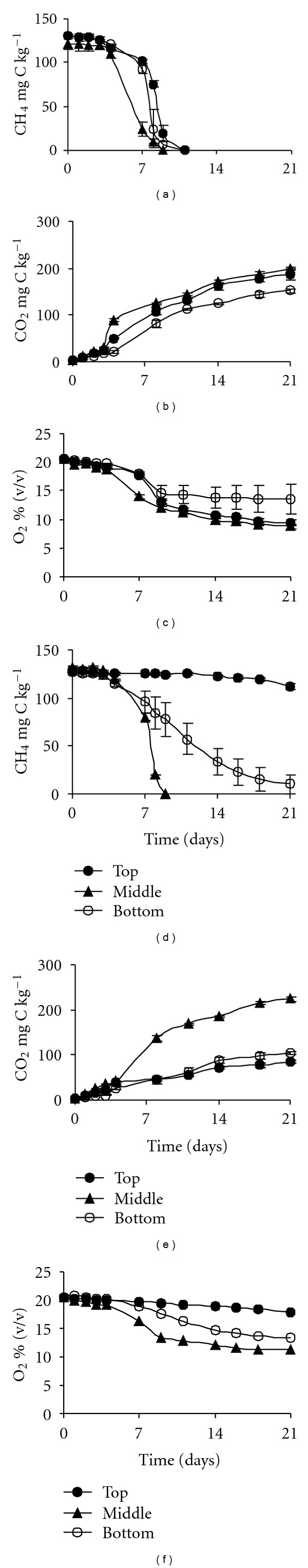
Changes of CH_4_, CO_2_, and O_2_ over time in loess soils collected from three slope positions and incubated with added methane, 5 kPa (methanotrophic potential). Top: summit, Middle: back slope, Bottom: bottom of the slope. Upper graphs (a–c) upper soil depth of 0–20 cm; lower graphs (d–f) lower soil depth of 20–40 cm. (a) and (d) cumulative CH_4_ production; (b) and (e) cumulative CO_2_ production; (c) and (f)—changes in O_2_ in the headspace. Points represent triplicate-means with standard error.

The capacity of CH_4_ consumption in the upper soils was slightly modified by the slope position (Figure 2(a)). All the soils collected at the 0–20 cm depth consumed completely the added methane (100%) after a 3-4 day lag. The highest methanotrophic activity was observed in the Middle soil, which rapidly utilized the whole gas between 4 and 9 day, at a rate of −20.66 mg CH_4_-C kg^−1^ d^−1^ (Table 3). CH_4_ consumption in the Top and Bottom samples lasted some longer, until the 11th incubation day (16.08 and 17.58 mg CH_4_-C kg^−1^ d^−1^, resp.). 

Deeper soil layers showed greater variation in CH_4_ oxidation capacity (Figure 2(d)). The highest activity was shown, again, by the soil collected from Middle position, which completely utilized 121.3 mg CH_4_-C kg^−1^ between the 3rd and 9th incubation days (at a rate of −12.26 mg CH_4_-C kg^−1^ d^−1^). The Bottom soil consumed 116.49 mg CH_4_-C kg^−1^ (92% of initial) at a rate of −7.95 mg CH_4_-C kg^−1^ d^−1^, whereas the Top soil oxidized only 14% of added CH_4_, at a low rate of −0.799 mg CH_4_-C kg^−1^ d^−1^ (*P* < 0.001) (Table 3). 

In general, CO_2_ production and O_2_ consumption followed the tendencies in CH_4_ uptake (Figure 2). The Middle soil of both depths showed an apparent increase in CO_2_, and a decrease in O_2_ in the headspace after 4 days of incubation, when CH_4_ oxidation started. Total CO_2_ evolution of 198.9 mg C kg^−1^ was, however, similar to that measured in the Top upper soil, which consumed significantly less CH_4_ (Table 3). The amount of CO_2_ evolved by the Middle upper soil was significantly higher than that produced in the Bottom soil (151.9 mg C kg^−1^, *P* < 0.001). Among the soils collected from deeper layers, the Middle soil produced more CO_2_ and consumed more O_2_ than the other soils, with accumulation of 224.1 mg CO_2_-C kg^−1^ and utilization of 13.6% (v/v) O_2_, *P* < 0.001 (Figure 2). 

### 3.3. Relations between Measured Soil Properties

**Figure 3:**
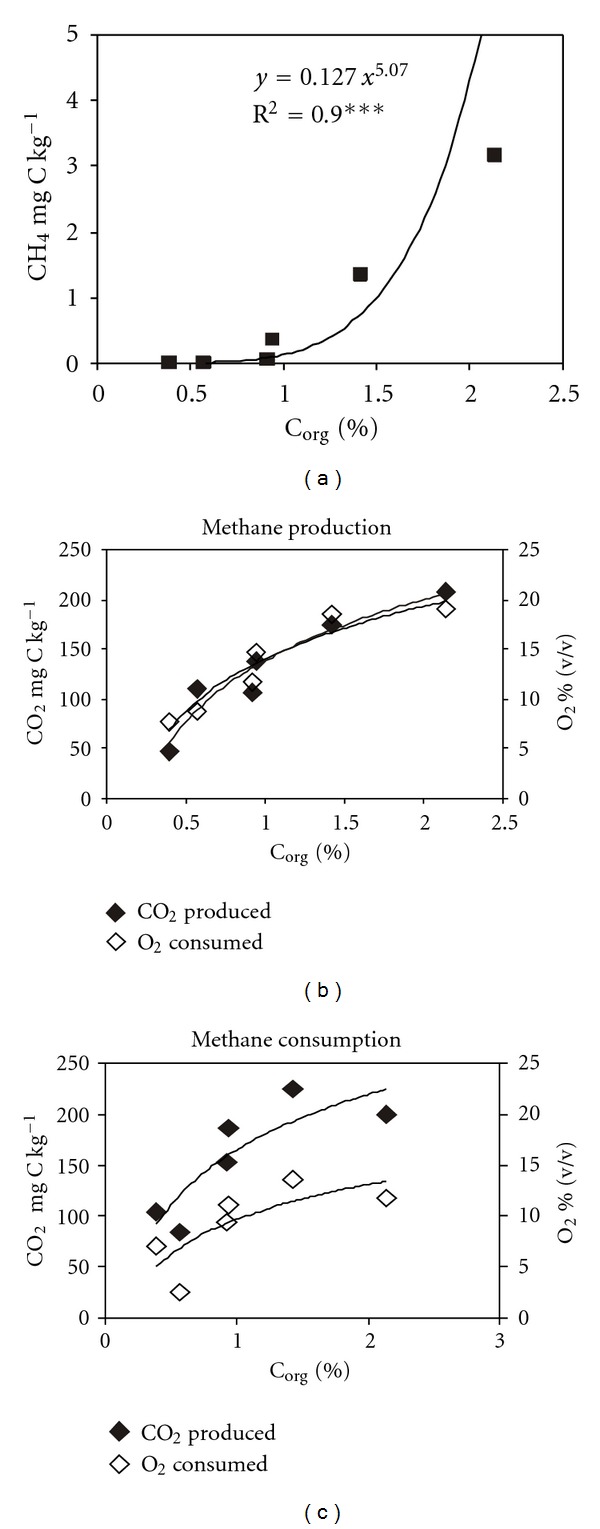
Relationships between total gases produced or consumed over time and organic carbon (C_org_) in soils collected from different slope positions. (a) CH_4_ produced in flooded soils versus C_org_; (b) CO_2_ produced and O_2_ consumed in flooded soils versus C_org_ (*y* = 7.60 · Ln(*x*) + 14.1, *R* ^2^ = 0.92***) and (*y* = 88.4 · Ln(*x*) + 138.7, *R* ^2^ = 0.93***); respectively, (c) CO_2_ produced and O_2_ consumed in wet soils enriched with CH_4_ soils versus C_org_ (*y* = 78.1 · Ln(*x*) + 165.2, *R* ^2^ = 0.74***) and (*y* = 4.38 · Ln(*x*) + 4.60, *R* ^2^ = 0.92*), respectively. Points present mean values. *** and *, *P* < 0.001 and *P* < 0.05, respectively.

The amount of methane produced in flooded soils showed a close relationship with the amount of organic C modified by the soil position in the slope (Figure 3(a)). 

Similar significant relations were observed for CO_2_ produced and O_2_ consumed during incubation of flooded and wet soils (i.e., in the course of methane production and oxidation, resp.) versus C_org_ (Figures 3(a) and 3(b)). Such correlations for methane oxidation were not shown. 

## 4. Discussion

Position in the landscape affects the accumulation and redistribution of water, nutrients, sediments, and organic matter. Soils on ridges and upper slopes will tend to loose soil and organic matter that will tend to accumulate on lower slopes and in depressions. Generally, soils in lower-slope positions will tend to have a wetter moisture regime for a longer period [33], while soil O_2_ concentrations may decrease significantly from ridges to valleys [34]. Methane emission from low-slope positions may be observed already one or three days after summer rainfall, depending on the intensity of precipitation [13]. It has been assumed that, in well-aerated soils, CH_4_ production in anaerobic microsites could be an important source of methane for methane oxidizing bacteria [35]. Little is known, however, about methane emission when usually well-drained soils become flooded for a short period [13]. The characteristics of CH_4_ oxidizing and producing communities and the factors which affect these characteristics as well as CH_4_ transport determine the magnitude of the surface CH_4_ flux to the atmosphere [36]. Our studies with loess soil collected from different slope positions and incubated under laboratory conditions showed that the slope position significantly affected the soil potential for CH_4_ production and oxidation and modified soil respiration involved in CH_4_ transformations. 

We compared soils from the Middle and Bottom positions with the soils at the summit (Top) position, which may be regarded as a reference soil. The methane production potential of the soil from the Middle position significantly increased (9-fold in the upper soil layer and even more in the deeper layer *P* < 0.001), while at the Bottom position CH_4_ production did not changed significantly (Table 2). In turn, the methane oxidation potential was unchanged in the 0–20 cm layer (as all upper soils depleted all added CH_4_) but strongly increased in the 20–40 cm soil layers and form the level of 14% in the Top soil, to 92–100% in the other soils (*P* < 0.05 or *P* < 0.001). 

In the course of methanogenesis, soil respiration underwent modification similar to that observed for the methanogenic potential. In the Middle slope position, CO_2_ production and O_2_ uptake were significantly stimulated as compared with the Top soil (by 50% and 30%, resp., *P* < 0.001 and *P* < 0.01). Soil collected at the Bottom position showed lower respiration than the Top soil (both CO_2_ and O_2_ less by about 20%, *P* < 0.01). In the deeper soil layer, the changes were generally more pronounced. 

Changes in soil respiration in the course of methanotrophy were apparently dependent on soil depth. In comparison with the Top site, the upper Middle and Bottom soils were not changed, as all soils consumed comparable amounts of methane. However, soils sampled from a depth of 20–40 cm respired at a significantly higher rate than the Top soil (up to 5 times) (*P* < 0.001). 

In the experiments of [21], water-stable aggregates were significantly different among landscape positions and decreased from lower > middle > summit landscape position. However, in some soils, the landscape effect was insignificant, for example, for enzyme activities or emission of CH_4_ and CO_2_ [19, 21]. Fang et al. [17] observed that neither potential net N mineralization nor nitrification was differentiated by the slope position, nor was accumulative emissions of N_2_O or CO_2_ from incubated soils in laboratory; in contrast, the ability to oxide CH_4_ appeared to decrease from the bottom to the top. 

It is well known that methane fluxes are strongly regulated by the presence or absence of methanotrophs (CH_4_ oxidizers), which are generally found in the upper (0–20 cm) soil [37]. On the other hand, methanogens (CH_4_ producers) use labile carbon compounds that were produced in the root zone and are less abundant with increasing distance from the soil surface, and rates of potential CH_4_ production decline with depth below the aerobic zone [38]. However, deeper soil also contributes to CH_4_ emission. In our experiment, both upper (0–20 cm) and lower (20–40 cm) soil layers of the Middle position evolved CH_4_. The process started relatively fast, after 17 days of flooded incubation at 25°C, and CH_4_ reached 3.16 mg CH_4_-C kg^−1^ (0.35% v/v of CH_4_ in the headspace) over 28-day incubation. Similarly, Mayer and Conrad [39] observed a rapid increase in CH_4_ production within 25 days of flooding an upland agricultural soil and a forest soil. It is possible that, in our experiment, CH_4_ emissions did not occur in the other soils since O_2_, which is the most thermodynamically favourable electron acceptor [29, 37], was still in the headspace. Methanogenesis is evidently inhibited by O_2_ this is apparent from field studies that show no overlap in the depth distributions of O_2_ penetration in soils or sediments and net CH_4_ production [38]. However, the lack of CH_4_ production in the presence of O_2_ *in situ* may be due to a combination of factors, of which O_2_ toxicity is just one. For example, methanogens are more sensitive to desiccation than O_2_ exposure in a paddy soil [40]. In our experiment, methane formation in the Middle soil was preceded by relatively rapid O_2_ consumption, which created soil hypoxia and allowed methanogens to develop. However, CH_4_ was also detected in the upper Top soil on day 21 (0.008% v/v in the headspace), although there was 8.4% (v/v) O_2_ in the headspace. 

Probably, the mechanisms of the slope position effect on methanogenic and methanotrophic activities are complex. Some decline in soil pH was observed for the Middle and Bottom soils as compared with the Top soil (from pH 8.1 to 7.6–7.9, Table 1). However, most methanogenic communities seem to be dominated by neutrophilic species [38]; in a survey of 68 methanogenic species conducted by Garcia et al. [41], most species grew best in a pH range from 6 to 8. Evidently, in our experiment, the soil position changed the sand, silt, and clay content at the depth of 20–40 cm (Table 1). The clay fraction content decreased from 6.3% in the Top soil to 4.2 and 4.3% in the Middle and Bottom soils, respectively. Similarly, the silt content decreased from 77.4% to 68.7 and 61.6%, respectively. A change in the sand content (an increase from 16.3% in the Top position to 27.5 and 34.5% in the Middle and Bottom positions, resp.) probably stimulated CH_4_ oxidation due to better draining and easier gas diffusion in soil containing more sand, because O_2_ is necessary for monooxygenase enzyme which catalyzes methane oxidation [42]. Coarse-textured soils have been documented as supporting CH_4_ oxidation by enhancing gas diffusion (CH_4_ and O_2_) into the soil [43]. 

Paluszek and Żembrowski [44] present their findings from a long-term study designed to explore effect of accelerated erosion on soil properties in a loessial landscape. They observed that slight, moderate, and severe erosion has an adverse effect on soil physical properties. The clay content and bulk density in Ap horizons of eroded soils are on the increase whereas the content of organic matter, content of water-stable aggregates, field water capacity, and retention of water useful for plants decrease. In the consequence, soil porosity, air capacity, and air permeability deteriorate. By contrast, in very severely eroded soils whose Ap horizons developed from carbonate loess, pore-size distribution, field water capacity and retention of water useful for plants are favourable and comparable to those in noneroded soils [44]. 

High organic C content in the Middle soil, as compared with the Top soil (Table 1), stimulated methane production under soil hypoxia. Probably, small differences in C_org_ between Top and Bottom soils may explain insignificant differences in methanogenic activity. Nevertheless, high correlation coefficients obtained for relationships between C_org_ and produced CH_4_, evolved CO_2_, and consumed O_2_ confirm their universal character (Figure 3). However, better explanation of the changes observed in our experiment needs more information on the properties of tested soils at different slope positions. 

## Figures and Tables

**Table 1:** Basic characteristics of loess soils at three slope positions.

Slope position	Soil depth (cm)	C_org_ (%)	C_inorg_ (%)	pH (H_2_O)	Sand	Silt	Clay
						(%)	
Top	0–20	0.97	0.690	8.11	33.6	62.1	4.3
	0–40	0.57	0.002	8.14	16. 2	77.4	6.3

Middle	0–20	2.14	0.006	7.60	27.1	68.7	4.2
	20–40	1.42	0.275	7.91	27.5	68.3	4.2

Bottom	0–20	0.92	0.657	7.88	30.9	64.9	4.2
	20–40	0.39	0.393	7.56	34.1	61.6	4.3

**Table 2:** CH_4_ production, CO_2_ evolution and O_2_ uptake of soils collected from three slope positions, and incubated for 28 days under flooding (average values ± standard error, *n* = 3).

Slope position	Soil depth (cm)	CH_4_ production		CO_2_ evolution (mg C kg^−1^)	O_2_ uptake % (v/v)
		Total (mg C kg^−1^)	Rate (mg C kg^−1^ d^−1^)		
Top	0–20	0.3595 ± 0.118	0.0316	138.1 ± 2.56	14.61 ± 0.85
	20–40	0.0017 ± 0.001	0.0001	110.2 ± 2.19	8.73 ± 0.04

Middle	0–20	3.1679*** ± 0.140	0.3042	207.6*** ± 2.34	19.14** ± 0.10
	20–40	1.3538*** ± 0.129	0.1162	174.6*** ± 0.48	18.49*** ± 0.29

Bottom	0–20	0.0584 ns ± 0.011	0.0065	106.7** ± 0.13	11.65** ± 0.12
	20–40	0.0018 ns ± 0.001	0.0003	47.8*** ± 0.59	7.71 ns ± 0.13

*, **, ***, different from the Top position (reference soil) at *P* < 0.05, *P* < 0.01 and *P* < 0.001, respectively, according to Student's *t*-test; ns—not significant difference.

**Table 3:** CH_4_ consumption, CO_2_ evolution, and O_2_ uptake in soils collected from three slope positions and incubated with 5 kPa methane for 21 days (average values ± standard error, *n* = 3).

Slope position	Soil depth (cm)	CH_4_ consumption			CO_2_ evolution (mg C kg^−1^)	O_2_ uptake % (v/v)
		Total (mg C kg^−1^)	% of initial CH_4_	Rate (mg C kg^−1^ d^−1^)		
Top	0–20	130.84 ± 2.31	100	−16.08	186.7 ± 11.4	11.14 ± 0.53
	20–40	17.80 ± 2.62	14	−0.799	83.9 ± 2.86	2.55 ± 0.11

Middle	0–20	121.36* ± 0.01	100	−20.66	198.9 ns ± 3.57	11.77 ns ± 0.34
	20–40	131.71* ± 0.80	100	−12.26	224.1*** ± 5.26	13.63*** ± 0.32

Bottom	0–20	130.96 ns ± 43.7	100	−17.58	151.9 ns ± 5.34	9.44 ns ± 0.38
	20–40	116.49*** ± 12.2	92	−7.954	104.1** ± 3.14	6.99*** ± 0.50

*, **, ***, different from the Top (reference) soil at *P* < 0.05, *P* < 0.01, and *P* < 0.001, respectively, according to Student's *t*-test; ns—not significant difference.
